# Factors associated with viral load testing and viral suppression among HIV-positive female sex workers in Nigeria

**DOI:** 10.1371/journal.pone.0304487

**Published:** 2024-05-31

**Authors:** Kene David Nwosu, Abiye Kalaiwo, Wingston Felix Ngambi, Janne Estill, Ughweroghene Kingston Omo-Emmanuel, Godwin Emmanuel, Olivia Keiser

**Affiliations:** 1 Institute of Global Health, University of Geneva, Geneva, Switzerland; 2 Office of HIV/AIDS and TB, US Agency for International Development, Abuja, Nigeria; 3 Department of Health Systems and Policy, Health Economics and Policy Unit, Kamuzu University of Health Sciences, Lilongwe, Malawi; 4 Office of HIV/AIDS and TB, US Agency for International Development (USAID), Abuja, Nigeria; 5 Heartland Alliance Nigeria, Abuja, Nigeria; United States Agency for International Development (USAID), NIGERIA

## Abstract

**Background:**

Female sex workers (FSWs) are at high risk for HIV infection and face unique barriers to receiving and adhering to testing and treatment. Early viral suppression and consistent viral load testing are critical to optimizing health and reducing transmission in this population. However, the factors associated with testing and successful viral suppression among FSWs are poorly understood, especially in Sub-Saharan Africa. Our study aimed to examine factors, including social, demographic, and clinical characteristics, associated with viral load testing and suppression among female sex workers initiating antiretroviral therapy in Nigeria.

**Methods:**

In this retrospective study, we analyzed routine programmatic data from FSWs enrolled in the National HIV Key Populations (KP) program in Nigeria. We included FSWs who were newly diagnosed with HIV and registered between January 2016 and January 2022. Primary outcomes of interest were a), receiving a viral load test at any point after treatment initiation and b), viral suppression (<1000 copies/ml) at the test closest to 6 months after treatment initiation. To identify factors associated with the outcomes of interest, we used univariable and multivariable logistic regression, with random intercepts for care facilities, and multiple imputation for missing values.

**Findings:**

Out of 34,976 FSWs, 97.1% (n = 33,945) received at least one viral load test, with 94.5% (n = 32,092) indicating viral suppression. The odds of receiving at least one viral load test were higher for those who entered treatment in more recent years, those with formal education and those with advanced HIV stages at baseline (adjusted odds ratios [aOR]: 1.17 [1.14–1.19] for those who entered treatment in 2020 vs. 2016; 1.02 [1.01–1.03] for post-secondary vs. no education; and 1.05 [1.01–1.10] for WHO clinical stage 3/4 vs. stage 1 respectively). The odds of successful viral suppression were higher for those who entered treatment in more recent years, but lower for those with advanced HIV stages at baseline (aOR: 1.13 [1.09–1.18] for 2022 vs. 2016; and 0.92 [0.87–0.98] for WHO clinical stage 3/4 vs. stage 1 respectively).

**Conclusions:**

The study underscored the relevance of timely diagnosis and ART initiation for optimal outcomes among HIV-positive FSWs in Nigeria. We also observed significant improvements in the likelihood of early viral load testing and suppression over the study period, reflecting advancements in the KP program. Further research should clarify factors driving these trends to further strengthen the HIV care pipeline for female sex workers.

## Introduction

Female sex workers (FSWs) face a disproportionately high burden of HIV globally, with prevalence rates that are sometimes up to 30 times greater than general populations [1,2]. Achieving viral suppression through antiretroviral therapy (ART) and consistent viral load testing is critical for effective HIV management in this key population [3–6]. However, FSWs encounter unique barriers that hinder their access and adherence to testing and treatment, including stigma, repressive legal environments, and mental health issues, and are at a high risk of HIV acquisition and transmission [5,6].

While factors influencing viral suppression and adherence to viral load monitoring have been extensively studied in people living with HIV (PLHIV) [3,4], less is known about the drivers of these outcomes specifically among FSWs. Notably few studies on this topic have been conducted in Sub-Saharan Africa, despite its large population of FSWs living with HIV, and much of the existing literature relies on small sample sizes or qualitative data alone [6,7].

Nigeria, the most populous country in Africa, has the fourth largest HIV epidemic globally, with FSWs disproportionately affected [8,9]. HIV prevalence rates as high as 21% have been reported among groups of FSWs in Nigeria [9], underscoring the need to optimize treatment outcomes in this population. Nigeria’s National HIV Key Populations (KP) program, which provides comprehensive HIV services to FSWs [10], presents an opportunity to understand barriers and facilitators to HIV care uniquely experienced by FSWs in a high-burden country.

The present study examines factors associated with the receipt of viral load tests and viral suppression among FSWs newly initiating ART in several Nigerian states. By identifying demographic, clinical, and structural factors associated with optimal viral load monitoring and viral suppression, the findings from this study can inform strategies to strengthen the HIV care continuum for FSWs in Nigeria and similar high-burden settings globally.

## Materials and methods

### Study setting

We used data from the National HIV Key Populations program in Nigeria, which provides prevention, care, and treatment services to key populations, which include female sex workers, men who have sex with men, transgender people, people who inject drugs, and people in incarceration. The program, which operates in seven states (Akwa Ibom, Bayelsa, Cross River, Jigawa, Lagos, and Niger), uses a venue-based sampling method that reaches FSWs across hotspots (brothels and non-brothel locations like streets, dance clubs, and bars) with a package of HIV prevention services, ART, and other ancillary interventions. At enrollment, FSWs confirmed to be HIV-positive are registered in free antiretroviral treatment programs in clinics and community care centers for key populations in Nigeria. Data about socio-demographic and clinical characteristics are collected, and participants receive refills, blood draws and other services either by visiting service delivery sites or through mobile field-level healthcare workers at specific centers.

At about 6 months and 12 months after beginning ART, participants take an initial and second viral load test to assess treatment effectiveness, following UNAIDS guidelines [11]. If viral suppression is achieved and maintained at both tests, they undergo annual tests after that. In cases where viral suppression is not achieved during the initial tests, participants undergo tests every 3–6 months after enhanced adherence counseling. Blood samples are shipped to designated national testing laboratories where they are tested. Viral load testing was done using Roche Cobas AmpliPrep/Cobas TaqMan (from 2015 until 2022), Abbott m2000sp and m2000rt (from 2015 till date), Roche Cobas 68/8800 (2019 till date), Hologic Panther (2021 till date) and Roche Cobas C4800 (2022 till date).

The study population included female sex workers newly diagnosed with HIV and enrolled in the Nigerian KP program between January 2016 and January 2022. Retrospective data were obtained from an electronic database used by the National HIV KP program to track services provided to female sex workers enrolled in the program. Included were individuals aged 18 years or older at program enrollment, who self-identified as having exchanged sex for money or other items of value, either regularly or occasionally in the 12 months prior to enrollment. These de-identified data were exported and provided in CSV tables to the study authors in August 2023 for analysis, under approval from the Federal Capital Territory Health Research Ethics Committee in Nigeria (approval no: FHREC/2023/01/127/20-07-23). Data were exported in September 2022 and accessed by the authors on August 2, 2023. Participants were required to have at least 9 months of potential follow-up, limiting the analysis to those enrolled up until January 2022. This criterion enabled the investigation of viral load tests conducted between 90- and 270-days post-ART initiation, a primary outcome of interest. Of note, viral load testing capacity was more limited in Nigeria during the early years of the treatment program (2016–2017) but expanded nationally over time.

### Outcomes and covariates

Our study examined two primary binary outcomes: 1) whether or not a participant received a viral load test at any point after ART initiation, and 2) whether they achieved viral suppression (viral load <1000 copies/mL) at the test closest to six months post-ART initiation.

In a supplementary analysis, we also assessed whether or not a received a viral load test specifically within the 90-to-270-day window after ART initiation. This aligns with the six-month testing guideline recommended by the WHO, but with a range of ± 3 months (180 days ± 90 days), in order to capture enough individuals with the outcome variable for robust analysis. Among those who received testing in the 90-to-270-day time window, we again assessed whether they achieved viral suppression. This time-restricted supplementary analysis was conducted to control for the amount of time under treatment and to focus on factors influencing suppression during this crucial window when the first test is recommended and around which viral suppression is typically first achieved [9].

For all outcomes, the following covariates were considered: age (categorized as 16–19, 20–29, 30–39, 40–49, 50–59, and 60+ years), highest educational level attained (none, primary, secondary, post-secondary), the state where services were received (Akwa Ibom, Bayelsa, Cross River, Jigawa, Lagos, Niger), marital status (never married, ever married), employment status (employed, not employed, student), body mass index (BMI) category (<18.5, 18.5–24.9, 25–30 or >30 kg/m^2^), systolic blood pressure (below 120, 120–129 and 130+ mmHg), WHO disease clinical stage at baseline (Stages 1 to 4), year of ART initiation (2016 to 2022), and the first ART regimen prescribed (tenofovir/lamivudine/dolutegravir [TFV-3TC-DTG], tenofovir/lamivudine/efavirenz [TFV-3TC-EFV], or other regimens).

### Statistical analysis

To evaluate the associations between predictor variables and the outcomes of interest (receipt of viral load testing and viral suppression), we carried out univariable and multivariable logistic regression analyses with random intercepts for care facilities, accounting for the variability across the 16 facilities. We addressed missing data in five variables (education, employment status, marital status, BMI category, and systolic blood pressure) through multiple imputation by chained equations, generating 25 imputed datasets, with all covariates and the outcome variable included in the imputation equations.

Each covariate was tested individually in a univariable logistic regression model to evaluate its association with the outcomes of interest: receipt of viral load testing and viral suppression. A Wald Chi-square test was then carried out on each univariable model to assess statistical significance, and covariates with p-values below a cut-off of 0.30 were considered potentially relevant and included in the multivariable models.

Statistical analyses were run in R version 4.2.2. Imputations were performed using the mice R package (version 3.15.0) and pooled univariable chi-square tests were performed with the miceadds R package (3.16.18).

### Ethical considerations

This analysis was conducted with routine data gathered through the national KP program. Study authors only accessed the de-identified data. Written informed consent was obtained for all participants in line with the Nigerian HIV testing services policy. Potential participants read (or were read) an information sheet (which is written in their local language) before providing consent. Ethical approval was obtained from the Federal Capital Territory, Health Research Ethics Committee, Nigeria (approval no: FHREC/2023/01/127/20-07-23).

### Funding

This study was supported by the Swiss National Science Foundation (grants no. 163878 and 320030_192452).

The funders of the study had no role in study design, data collection, data analysis, data interpretation, or writing of the report.

## Results

### Sample characteristics

**Fig 1:**
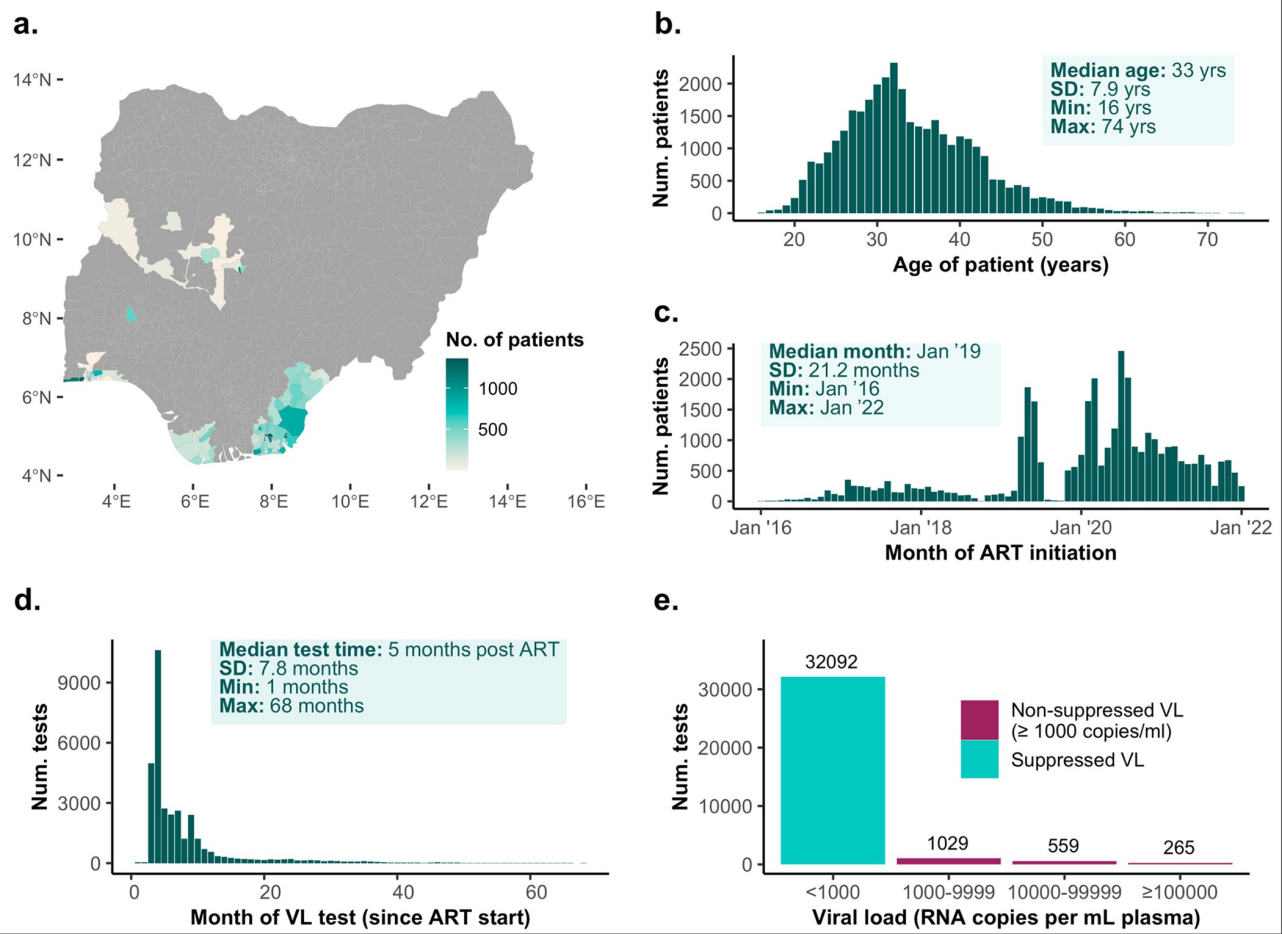
Sample characteristics and viral load results. (a) Choropleth map showing the distribution of participants by local government area (LGA) of residence. (b) Age (years) distribution of participants at ART initiation. (c) Time distribution of ART initiation. (d) Time distribution of index viral load tests. For each respondent with more than one test, the test nearest to 6 months post ART was selected as an index test (e) Viral load levels for index tests.

The cross-sectional analysis included 34,976 female sex workers newly diagnosed with HIV and initiating ART between January 1, 2016 and January 18, 2022, across seven states in Nigeria (Table **1 and** Fig **1**). The median follow-up time was 2.1 years, with a total of 78,700 person-years of follow-up. Most participants (76%, n = 26,599) were aged between 20 and 39 years. Approximately 60% had secondary education as their highest formal education level (n = 20,403) and about half reported no formal employment outside of sex work (43%, n = 15,031). Most participants resided in Akwa Ibom (39%, n = 13,583) and Lagos state (25%, n = 8,800) and nearly nine in ten (87%, n = 30,569) had never been married. Most participants started ART in 2020 (44%, n = 15,386), and 2021 (22%, n = 7,854) reflecting an expansion of the Nigerian HIV KP program at that time.

**Table 1:** Characteristics of the study sample.

Characteristic	N	Percent
Total	34,976	-
**Age group (years)**		
16–19	223	0.6
20–29	10,541	30.1
30–39	16,058	45.9
40–49	6,722	19.2
50–59	1,240	3.5
60+	192	0.5
**Education**		
None	1,852	5.3
Primary	3,588	10.3
Secondary	20,403	58.3
Post Secondary	7,740	22.1
Missing education	1,393	4
**Employment status**		
Employed	17,340	49.6
Not employed	15,031	43
Student	2,015	5.8
Missing employment status	590	1.7
**State of residence**		
Akwa Ibom	13,583	38.8
Bayelsa	1,990	5.7
Cross River	8,091	23.1
Lagos	8,800	25.2
Niger	2,510	7.2
Ogun	2	0
**Marital status**		
Never married	30,569	87.4
Ever married	4,166	11.9
Missing marital status	241	0.7
**ART start year**		
2016	588	1.7
2017	2,686	7.7
2018	1,510	4.3
2019	6,706	19.2
2020	15,386	44
2021	7,854	22.5
2022	246	0.7
**WHO baseline clinic stage**		
Stage I	33,895	96.9
Stage II	1,023	2.9
Stage III & IV	58	0.2
**BMI category (kg/m^2^)**		
Below 18.5 (Underweight)	1,385	4
18.5–24.9	21,341	61
25–30 (Overweight)	7,410	21.2
30+ (Obese)	2,059	5.9
Missing bmi category	2,781	8
**First ART regimen**		
Other regimen	63	0.2
TDF-3TC-DTG	29,883	85.4
TDF-3TC-EFV	5,030	14.4
**Systolic blood pressure (mmHg)**		
Below 120 (Normal)	18,780	53.7
120–129 (Elevated)	12,509	35.8
130+ (Hypertensive)	2,727	7.8
Missing systolic blood pressure	960	2.7

Abbrevations: N, number of individuals in each stratum. BMI: Body mass index. ART: Anti-retroviral therapy.

At baseline, nearly all participants (97%, n = 33,895) were in WHO HIV clinical stage 1. About two-thirds (61%, n = 21,341) had a normal body mass index (18.5–24.9 kg/m2), while 21% (n = 7,410) were overweight (25–29.9 kg/m2) and 6% (n = 2,059) were obese (30+ kg/m2). Over a third of individuals had elevated (120–129 mmHg) systolic blood pressure (36%, n = 12,509) and 8% (n = 2,727) had hypertensive (130+mmHg) systolic pressure. Most participants (85%, n = 29,883) were placed on TDF-3TC-DTG (tenofovir disoproxil fumarate, lamivudine, and dolutegravir) ART regimen at baseline, with 13% (n = 5,030) prescribed the TDF-3TC-EFV (tenofovir disoproxil fumarate, lamivudine, and efavirenz) regimen.

### Viral load testing

Among the 34,976 female sex workers, 89,834 viral load tests were carried out. Nearly all participants (97.1%, n = 33,945) received an initial viral load test during the study period, and a majority of participants (63.8%, n = 22,321) received a test in the 90-to-270-day period after ART initiation.

**Fig 2:**
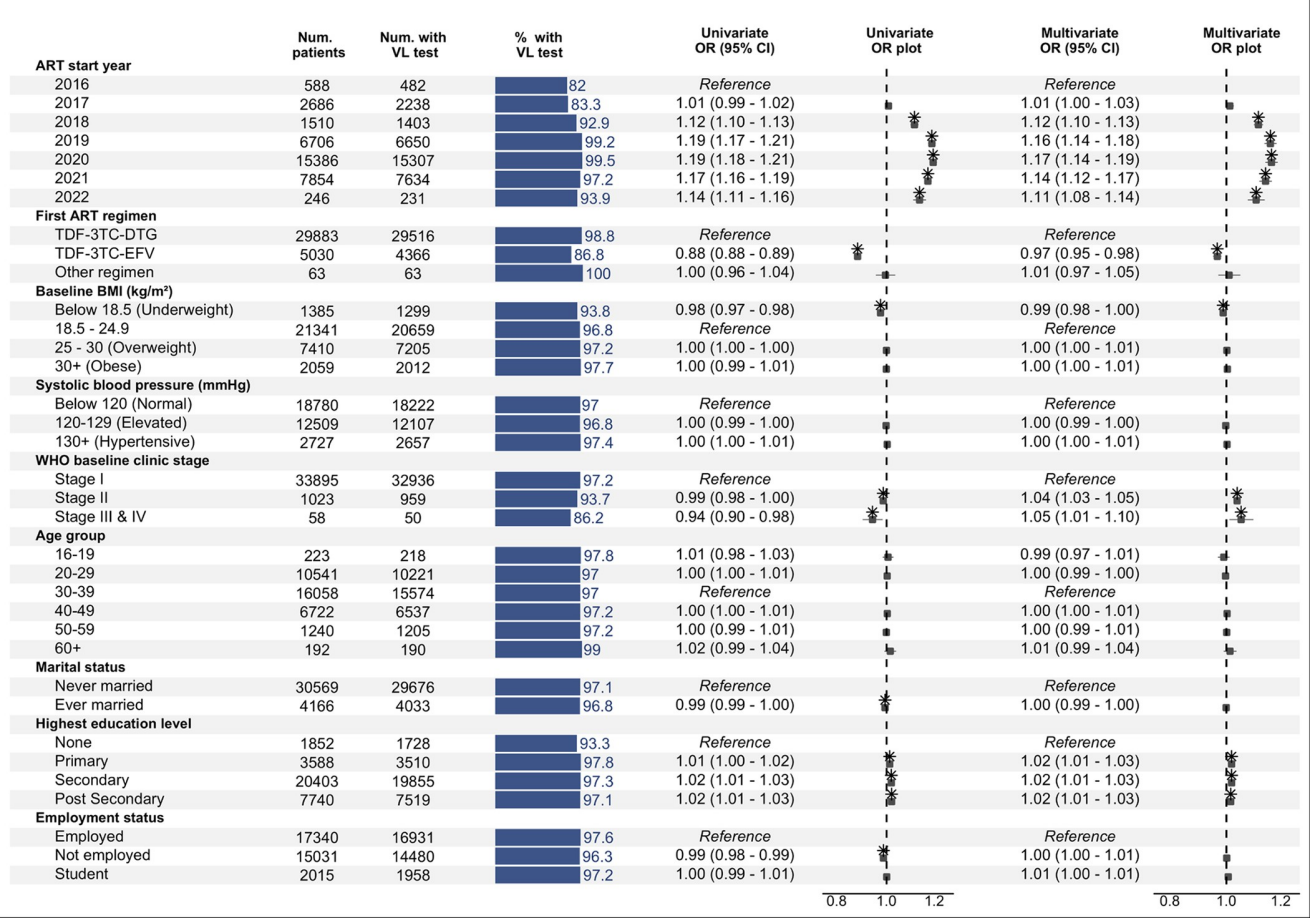
Factors associated with ever receiving a viral load test among FSWs in the Nigerian KP HIV treatment program. Based on logistic regression analysis with random intercepts for the treatment/testing facility. Box-whisker plots indicate odds ratios and 95% confidence intervals. Asterisks indicate statistical significance at a 0.05 alpha level. VL test: Viral load test. BMI: Body mass index, OR: Odds ratio, CI: Confidence interval.

Univariable and multivariable analyses revealed a temporal trend in the odds of ever receiving a viral load test (Fig 2). Compared to those initiating ART in 2016, the adjusted odds of being tested increased for those initiating ART from 2017 to 2020, ranging from a non-significant 1% higher odds for those starting ART in 2017 (aOR 1.01 [1.00–1.03]) to 17% higher odds for those starting ART in 2020 (aOR 1.17 [1.14–1.19]). This change likely reflects the increasing availability of viral load testing services in Nigeria over the study period.

ART regimens and WHO clinical stages were associated with the odds of testing. Participants on the TDF-3TC-EFV regimen had lower odds of testing than those on TDF-3TC-DTG (aOR 0.97 [0.95–0.98]). The association between baseline WHO disease clinical stage and the odds of viral load testing showed a complex pattern: unadjusted analysis indicated lower odds of testing for those at more advanced clinical stages (2–4) relative to stage 1, but the adjusted analysis reversed the relationship: higher adjusted odds of testing were seen for FSWs at stage 2 (aOR 1.04 [1.03–1.05]) or stage III/IV (aOR 1.05 [1.01–1.10]) compared to those at stage 1.

In addition to clinical factors, educational level was also associated with viral load testing, though the magnitude of the association was low. Participants with any level of formal education (primary, secondary, or post-secondary) were significantly more likely to undergo viral load testing than those with no education (aOR 1.02 [1.01–1.03], aOR 1.02 [1.01–1.03], and 1.02 [1.01–1.03], respectively).

**Fig 3:**
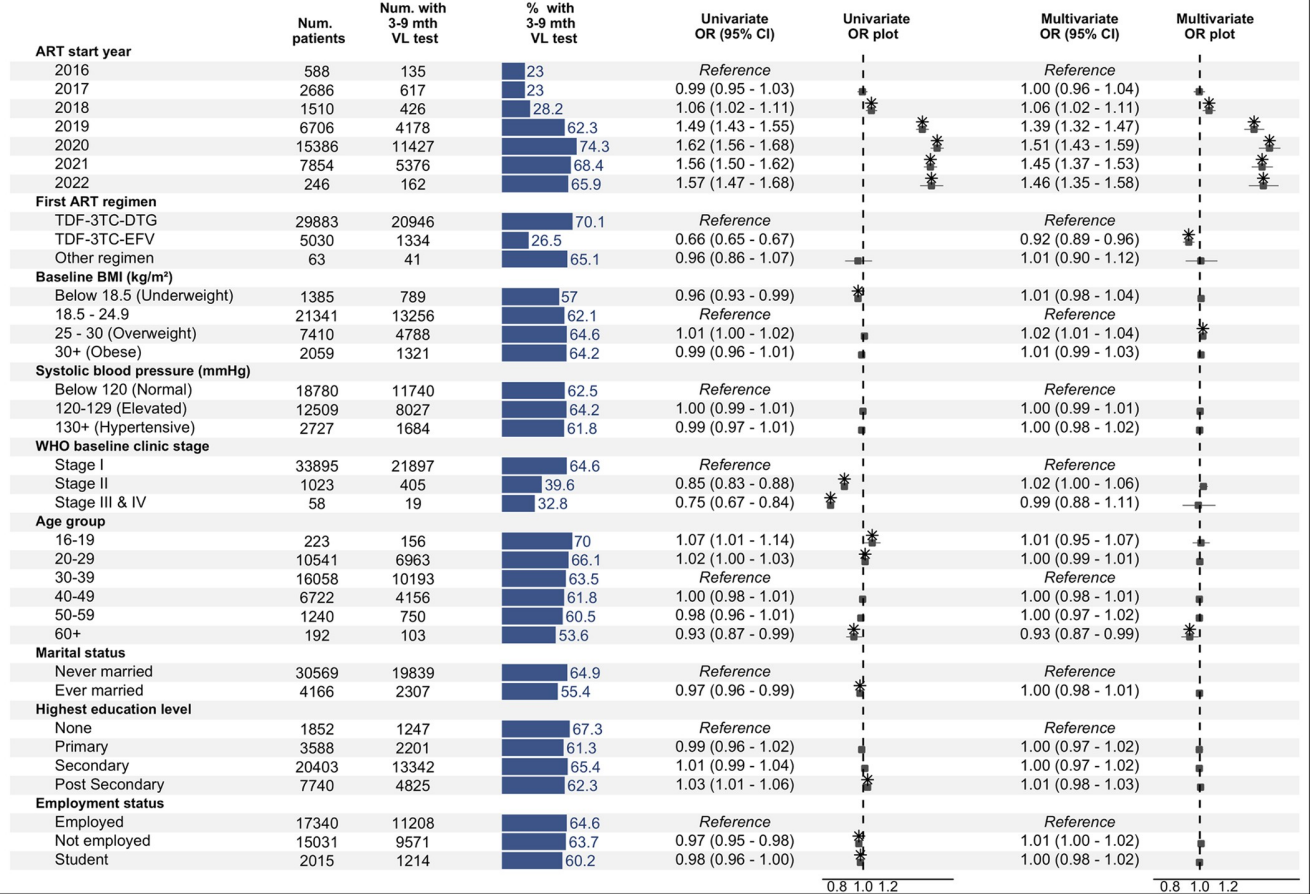
Factors associated with receiving a viral load test 90–270 days after ART initiation among Nigerian female sex workers. Based on logistic regression analysis with random intercepts for the treatment/testing facility. Box-whisker plots indicate odds ratios and 95% confidence intervals. Asterisks indicate statistical significance at a 0.05 alpha level. VL test: Viral load test. BMI: Body mass index, OR: Odds ratio, CI: Confidence interval.

The supplementary analysis examining rates of testing between 90 and 270 days after ART initiation found similar significant trends for regimen and ART start year (Fig 3).

### Viral suppression

To determine factors associated with viral suppression among participants with multiple viral load tests, we selected the test result closest to 6 months on ART as the index test for each participant. The distribution of these index tests is shown in Fig 1D. Among the 33,945 index tests, viral suppression (< 1000 copies per mL) was achieved for 94.5% (n = 32,092; Fig 1E).

**Fig 4:**
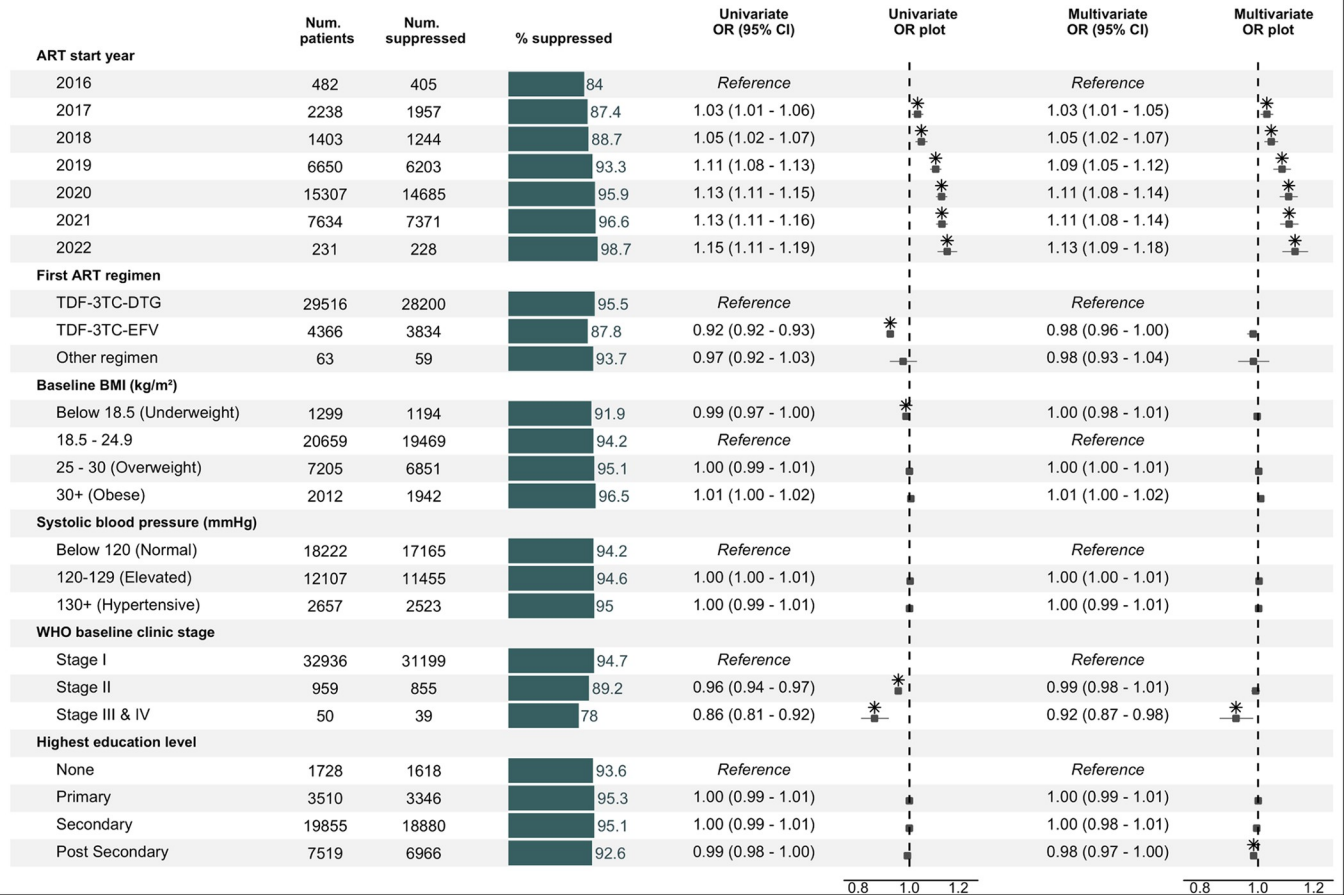
Factors associated with viral suppression among FSWs in the Nigerian KP HIV treatment program. For individuals with multiple tests, the test closest to 6-months post ART was used as the index test for determining viral suppression. Based on logistic regression analysis with random intercepts for the treatment/testing facility. Box-whisker plots indicate odds ratio and 95% confidence interval. Asterisks indicate statistical significance at a 0.05 alpha level. BMI: Body mass index, OR: Odds ratio, CI: Confidence interval.

Univariable and multivariable logistic regression analysis revealed that the odds of viral suppression increased for those starting ART in later years (Fig 4). Compared to those who initiated ART in 2016, the adjusted odds of suppression were 3% higher for those who started in 2017 (aOR 1.03 [1.01–1.05]) and 13% higher for those who started in 2022 (aOR 1.13 [1.09–1.18]).

Additionally, a significant relationship was observed with baseline clinical stage—compared to those who initiated ART at stage 1 of disease, the odds of suppression were 1% lower for those initiating at stage 2 (aOR 0.99 [0.98–1.01]) and 8% lower still for those who initiated ART at stage 3/4 (aOR 0.92 [0.87–0.98]). Finally, ART regimen was significantly associated with suppression in the univariable analysis, with tenofovir/lamivudine/efavirenz linked to lower odds than tenofovir/lamivudine/dolutegravir (univariate OR 0.92 [0.92–0.93]), but this relationship was not significant after adjustment for other factors (aOR 0.98 [0.96–1.00]).

**Fig 5:**
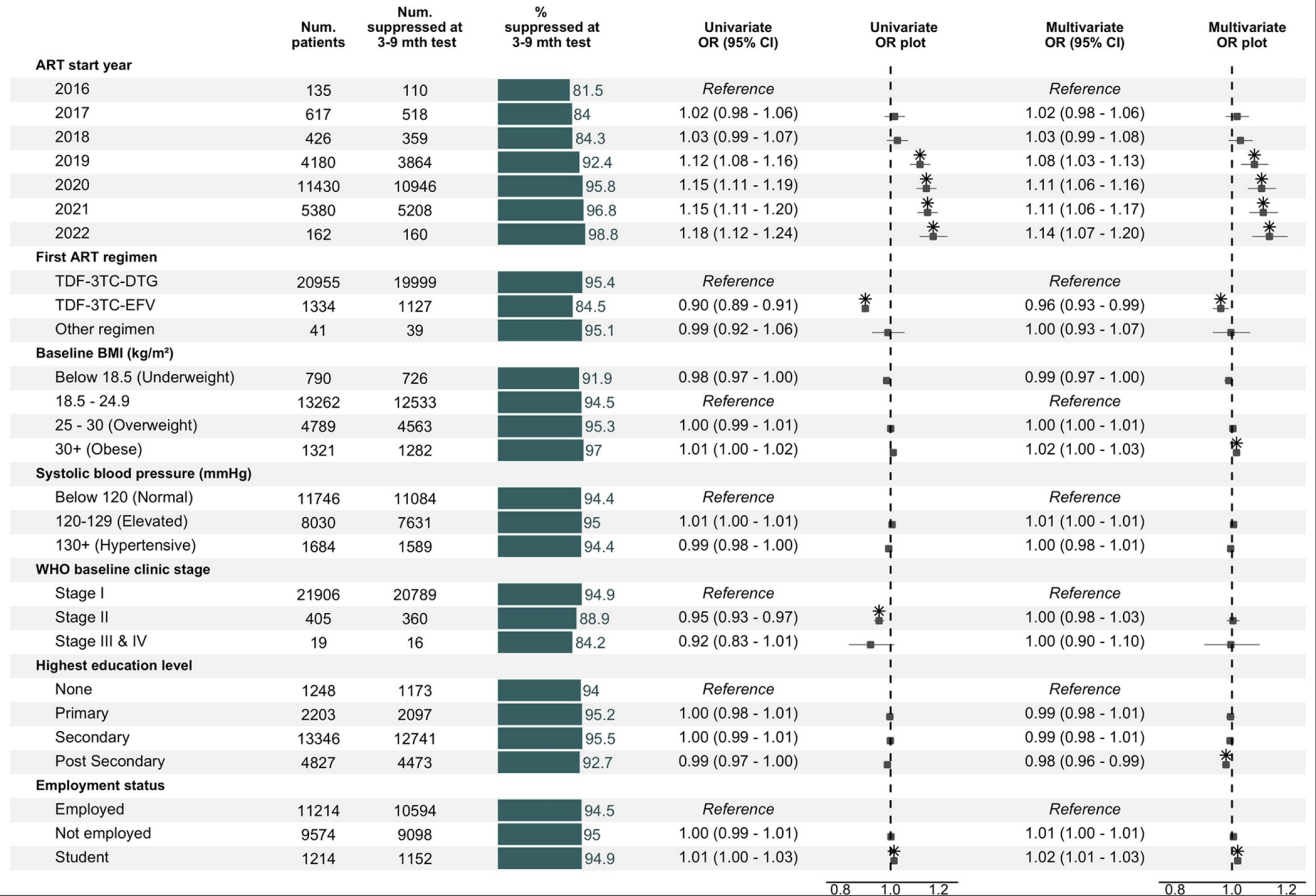
Factors associated with viral suppression among FSWs tested between 90 and 270 days after ART initiation. Based on logistic regression analysis with random intercepts for the treatment/testing facility. Box-whisker plots indicate odds ratio and 95% confidence interval. Asterisks indicate statistical significance at a 0.05 alpha level. BMI: Body mass index, OR: Odds ratio, CI: Confidence interval.

In the supplementary analysis examining viral suppression only among participants tested between 90 and 270 days after ART initiation, similar trends were observed with the variables ART start year, baseline BMI, education level, and WHO baseline clinical stage (Fig 5).

## Discussion

Our study investigated factors associated with receipt of viral load testing and subsequent viral suppression among FSWs newly initiating ART in Nigeria using a large-scale analysis of data from the National HIV Key Populations program. Overall, we found high rates of viral load testing and viral suppression, with 97% of participants receiving a viral load test over the study period, and 94.5% of participants virally suppressed at the test closest to 6 months.

The temporal trend observed in the study—with increasing likelihood of viral testing and viral suppression for those who started treatment in more recent years—could be attributable to improvements in the program’s reach and the provision of care over time. This finding is consistent with global trends, where increased access to ART, better follow-up services, and improved treatment regimens have contributed to better HIV outcomes [12].

Our results also revealed a relationship between education and odds of viral load testing, with those who completed any level of formal education having higher odds of testing than those with no education. A similar result was seen in a study in Bangladesh, which found that FSWs with secondary or higher levels of education were significantly more likely than others to have been tested for HIV [13]. These findings underscore the need to promote education among FSWs and the general population, to improve access and adherence to healthcare services [14].

We found an association between a more advanced WHO clinical stage at baseline and lower odds of viral suppression. This association is well documented across HIV-positive populations, including key populations [3,4], as antiretrovirals are less effective at advanced stages. This association should be interpreted with caution since WHO staging provides a subjective measure of disease progression compared to more objective CD4 count data, which was unavailable in this dataset. Nonetheless, considering the overall evidence from other studies, this finding points to the importance of early HIV testing, diagnosis, and treatment initiation among FSWs to maximize viral suppression. Targeted interventions addressing barriers to early diagnosis and care, such as stigma, discrimination, poor healthcare infrastructure, and limited HIV testing, may benefit this population [15,16].

Our study observed that individuals with higher education levels had marginally lower odds of viral suppression than those with less education. This is a counter-intuitive finding since education is often positively correlated with health outcomes and access to healthcare services [14]. However, the effect size of this association is relatively small—1% lower odds—indicating that the observed relationship might be attributable to random fluctuations and multiple hypothesis testing.

There are several limitations to our study. First, the observational design precludes drawing conclusive causal inferences between the factors investigated and the outcomes of interest. Second, our study relied on routinely collected programmatic data, rather than data collected in the course of our research study, and therefore may be subject to errors and inconsistencies in data quality of which we are not aware [17]. Third, our analysis may have been affected by unmeasured confounding factors, such as individual-level socioeconomic status or healthcare access, which were not available in the dataset. Fourth, since CD4 count data were not available for the majority of individuals, we relied on WHO clinical staging to classify HIV disease severity, which is less accurate than CD4 counts for estimating severity. Despite these limitations, the large sample size and robust statistical analyses provide valuable insights into factors influencing the cascade of care among FSWs in Nigeria.

Overall, this study confirms that early HIV diagnosis and access to care among female sex workers is key to maximizing treatment outcomes [18,19]. It also points to a possible effect of female education in improving outcomes among high HIV burden key populations. In addition, the increasing likelihood of viral load testing and viral suppression over time is promising and indicates that persistent efforts to improve HIV care and treatment for female sex workers in Nigeria are succeeding. However, the factors driving these improvements over time are not well understood. Further research, including in-depth qualitative studies with both FSWs and health service providers, could help identify the key drivers contributing to these positive changes. This would provide a more holistic understanding of how to improve HIV care for FSWs, ultimately reducing HIV incidence and improving HIV outcomes among FSWs and the broader population. In addition, research utilizing more objective measures of HIV disease status such as CD4 counts, rather than just clinical staging, would strengthen the analysis and conclusions related to the relationship between disease severity and viral suppression.

## Supporting information

S1 Fig Associated data for all tables and figures. Microsoft Excel Spreadsheet with Supporting Data. Each sheet contains the data pertaining to a specific figure or subfigure.
(XLSX)

## References

[1] Abdella S, Demissie M, Worku A, Dheresa M, Berhane Y. HIV prevalence and associated factors among female sex workers in Ethiopia, east Africa: A cross-sectional study using a respondent-driven sampling technique. *eClinicalMedicine* 2022; 51: 101540. doi: 10.1016/j.eclinm.2022.101540 35813094 PMC9256839

[2] Baral S, Beyrer C, Muessig K, et al. Burden of HIV among female sex workers in low-income and middle-income countries: a systematic review and meta-analysis. *The Lancet Infectious Diseases* 2012; 12: 538–49. doi: 10.1016/S1473-3099(12)70066-X 22424777

[3] Gardner EM, McLees MP, Steiner JF, Rio C del, Burman WJ. The Spectrum of Engagement in HIV Care and its Relevance to Test-and-Treat Strategies for Prevention of HIV Infection. *Clinical Infectious Diseases* 2011; 52: 793–800. doi: 10.1093/cid/ciq243 21367734 PMC3106261

[4] Heestermans T, Browne JL, Aitken SC, Vervoort SC, Klipstein-Grobusch K. Determinants of adherence to antiretroviral therapy among HIV-positive adults in sub-Saharan Africa: a systematic review. *BMJ Global Health* 2016; 1: e000125. doi: 10.1136/bmjgh-2016-000125 28588979 PMC5321378

[5] Shannon K, Strathdee SA, Goldenberg SM, et al. Global epidemiology of HIV among female sex workers: influence of structural determinants. *The Lancet* 2015; 385: 55–71. doi: 10.1016/S0140-6736(14)60931-4 25059947 PMC4297548

[6] Nnko S, Kuringe E, Nyato D, et al. Determinants of access to HIV testing and counselling services among female sex workers in sub-Saharan Africa: a systematic review. *BMC Public Health* 2019; 19. doi: 10.1186/s12889-018-6362-0 30611219 PMC6321716

[7] Lancaster KE, Cernigliaro D, Zulliger R, Fleming PF. HIV care and treatment experiences among female sex workers living with HIV in sub-Saharan Africa: A systematic review. *African Journal of AIDS Research* 2016; 15: 377–86. doi: 10.2989/16085906.2016.1255652 27974017 PMC5541376

[8] Joint United Nations Programme on HIV/AIDS. Country progress report—Nigeria [Internet]. Geneva: UNAIDS; 2020 [cited 2023 Sep 25]. Available from: https://www.unaids.org/sites/default/files/country/documents/NGA_2020_countryreport.pdf.

[9] Okafor UO, Crutzen R, Ifeanyi O, Adebajo S, Van den Borne H. HIV prevalence and high-risk behaviour of young brothel and non-brothel based female sex workers in Nigeria. *BMC Res Notes*. 2017; 10:380. doi: 10.1186/s13104-017-2712-8 28797278 PMC5553858

[10] Onovo A, Kalaiwo A, Katbi M, Ogorry O, Jaquet A, Keiser O. Geographical Disparities in HIV Seroprevalence Among Men Who Have Sex with Men and People Who Inject Drugs in Nigeria: Exploratory Spatial Data Analysis. JMIR Public Health Surveill. 2021 May; 7(5):e19587. doi: 10.2196/19587 34028360 PMC8185612

[11] World Health Organization. Consolidated guidelines on the use of antiretroviral drugs for treating and preventing HIV infection: recommendations for a public health approach [Internet]. 2nd ed. Geneva: World Health Organization; 2016. Available from: https://iris.who.int/bitstream/handle/10665/208825/97892?sequence=1. 27466667

[12] Bekker L-G, Alleyne G, Baral S, et al. Advancing global health and strengthening the HIV response in the era of the Sustainable Development Goals: the International AIDS SocietyLancet Commission. *The Lancet* 2018; 392: 312–58. 10.1016/S0140-6736(18)31070-5 PMC6323648 30032975

[13] Hossain G, Uddin SM, Islam A, et al. Factors Associated with HIV Testing Behaviors among Female Sex Workers in Rajshahi City, Bangladesh. *Journal of Life Sciences* 2017; 9: 104–10.

[14] Faust L, Yaya S. The effect of HIV educational interventions on HIV-related knowledge, condom use, and HIV incidence in sub-Saharan Africa: a systematic review and meta-analysis. *BMC Public Health* 2018; 18. doi: 10.1186/s12889-018-6178-y 30424761 PMC6234686

[15] Decker MR, Crago A-L, Chu SKH, et al. Human rights violations against sex workers: burden and effect on HIV. *The Lancet* 2015; 385: 186–99. doi: 10.1016/S0140-6736(14)60800-X 25059943 PMC4454473

[16] Logie CH, James Ll, Tharao W, Loutfy MR. HIV, Gender, Race, Sexual Orientation, and Sex Work: A Qualitative Study of Intersectional Stigma Experienced by HIV-Positive Women in Ontario, Canada. *PLoS Medicine* 2011; 8: e1001124. doi: 10.1371/journal.pmed.1001124 22131907 PMC3222645

[17] Gloyd S, Wagenaar BH, Woelk GB, Kalibala S. Opportunities and challenges in conducting secondary analysis of HIV programmes using data from routine health information systems and personal health information. *Journal of the International AIDS Society* 2016; 19: 20847. doi: 10.7448/IAS.19.5.20847 27443274 PMC4956739

[18] Kitahata MM, Gange SJ, Abraham AG, et al. Effect of Early versus Deferred Antiretroviral Therapy for HIV on Survival. *New England Journal of Medicine* 2009; 360: 1815–26. doi: 10.1056/NEJMoa0807252 19339714 PMC2854555

[19] Boyd M, Boffito M, Castagna A, Estrada V. Rapid initiation of antiretroviral therapy at HIV diagnosis: definition, process, knowledge gaps. *HIV Medicine* 2019; 20: 3–11. doi: 10.1111/hiv.12708 30724450

